# Update on HIV pandemic

**DOI:** 10.1186/1742-4690-9-S1-I4

**Published:** 2012-05-25

**Authors:** Anna Mia Ekstrom

**Affiliations:** 1 Karolinska Institutet, Stockholm, Sweden

## 

The aim of this presentation is to present the state of the HIV pandemic. The talk will start with an overview of current trends in HIV prevalence and incidence, mode of transmission, and, access to prevention and treatment in various regions of the world. Thereafter, the presentation will focus on three main dilemmas related to the interpretation of HIV prevalence, treatment coverage and recent policy guidelines for HIV treatment and prevention. The first dilemma to be discussed is the difficulty in measuring HIV incidence using current methodologies. HIV prevalence has become a poor estimate of HIV incidence given the varying and rapidly changing access to antiretroviral treatment (ART) as well as retention in ART programs affecting both survival and incidence. The dilemma of selecting an appropriate population group for monitoring HIV trends will also be mentioned. Neither antenatal clinic measurements of point prevalence rates or demographic health surveillance techniques are free of bias. Secondly, the dilemma of using coverage measures to assess the success of scaling up access to ART and prevention of mother to child transmission (PMTCT) will also be discussed. The problem of identifying an appropriate denominator for a lethal disease is one problem. Another one is the high drop-out from both ART and PMTCT programs, making reported coverage estimates based on enrollment, rather than completion, hard to interpret. Finally some recent policies related to HIV treatment and prevention such as Test and Treat, couple testing and the new WHO guidelines for PMTCT will also be briefly discussed in relation to country GDP and health systems capacity.

